# The Contributions of Language Skills and Comprehension Monitoring to Chinese Reading Comprehension: A Longitudinal Investigation

**DOI:** 10.3389/fpsyg.2021.625555

**Published:** 2021-03-19

**Authors:** Aiping Zhao, Ying Guo, Shuyan Sun, Mark H. C. Lai, Allison Breit, Miao Li

**Affiliations:** ^1^School of Foreign Languages and Literatures, Shandong University, Jinan, China; ^2^School of Education, University of Cincinnati, Cincinnati, OH, United States; ^3^Department of Psychology, University of Maryland, Baltimore, Baltimore, MD, United States; ^4^Department of Psychology, University of Southern California, Los Angeles, CA, United States; ^5^Department of Curriculum and Instruction, University of Huston, Houston, TX, United States

**Keywords:** comprehension monitoring, language skills, reading, Chinese, longitudinal

## Abstract

This study examined how vocabulary, syntactic knowledge, and orthographic knowledge are related to comprehension monitoring and whether comprehension monitoring mediates the relations between these language skills and reading comprehension. Eighty-nine Chinese children were assessed on their vocabulary, syntactic knowledge, orthographic knowledge, and comprehension monitoring in Grade 1. Their reading comprehension skills were assessed in Grade 1 and Grade 3. Results showed that in Grade 1, comprehension monitoring mediated the relations between vocabulary and syntactic knowledge and reading comprehension. For Grade 3 reading comprehension, syntactic knowledge in Grade 1 was the only significant predictor. These findings indicate that multiple language skills make direct and indirect contributions *via* comprehension monitoring to Chinese reading comprehension, and the relations would change as children’s reading skills develop.

## Introduction

Successful reading comprehension involves the active building of a situation model, i.e., a coherent mental representation of the text. According to the construction-integration model of reading, text comprehension is composed of three sub-processes: surface structure, textbase representation, and situation model (Kintsch, 1988; Kintsch and Rawson, 2007; McNamara and Magliano, 2009; Kendeou et al., 2014). The building of surface structure refers to the deriving of meaning from printed words and phrases, while textbase representation involves the building of propositions. Once the initial propositions are built, they are integrated across sentences and with prior knowledge for the construction of a situation model. Multiple skills, including both language and cognitive skills, contribute to the three sub-processes of reading (Hannon and Daneman, 2001; Perfetti et al., 2007; Oakhill, 2020). Specifically, language skills, including phonological knowledge, morphological knowledge, vocabulary knowledge, and orthographic knowledge, are important word-level skills in the process of building surface structure (Ehri, 2014; Quinn et al., 2015; Xie and Wu, 2019; Liu and Liu, 2020). In the textbase representation process, the language skill of syntactic knowledge is essential as it helps readers to group words, trace the logical and semantic relations among linguistic constituents, and build initial propositions (So and Siegel, 1997; Cheung et al., 2007; Brimo et al., 2018; Simpson et al., 2020). In the situation model construction process, cognitive skills, such as comprehension monitoring and inference making, are necessary to detect internal and external inconsistences and draw inferences (Cain et al., 2004; Connor et al., 2015; Oakhill et al., 2015; Wong et al., 2017). As such, language skills might not only have direct effect on reading by allowing readers to build initial propositions but also indirect effect on reading by facilitating readers’ engagement in comprehension monitoring, which is a higher level cognitive skill built on foundational language skills (Kintsch, 1988; Perfetti et al., 2007; Zargar et al., 2020). That is, the language and cognitive skills that underlie the three sub-processes of reading might have hierarchical and dynamic relations among them (Cromley and Azevedo, 2007; Kim, 2015, 2016, 2017, 2020).

Dynamic relations indicate that the relative importance of different component skills in predicting reading comprehension might vary as a function of development (Saarnio et al., 1990; Cain et al., 2004). At the early phase of reading development, word-level processes (e.g., orthographic knowledge and vocabulary) take up a large proportion of children’s processing capacity, with little left for sentence or text comprehension processes (Perfetti, 1985; Hannon and Daneman, 2001; McNamara and Magliano, 2009). As children’s word-level processes become more fluent and automatized through time and experience, more processing capability is freed up for processes at sentence and text levels (e.g., syntactic knowledge and inference making; Vellutino et al., 2007; Garcia and Cain, 2014). As an important cognitive skill, comprehension monitoring might also make greater contribution to reading as children become more cognitively advanced. LARRC and Yeomans-Maldonado (2017) found that among English-speaking children their comprehension monitoring at Grade 1 predicted reading at Grade 3 but vocabulary at Grade 1 did not.

Despite the growing evidence supporting the hierarchical relations among component skills of reading (e.g., vocabulary, syntactic knowledge, comprehension monitoring, and inference; Cromley and Azevedo, 2007; Kim, 2015, 2016, 2020), there are two critical gaps in this line of research. First, previous studies focused on the direct and mediated relations of language and cognitive skills to reading comprehension of children speaking languages with alphabetic orthography (i.e., English and Korean). The hierarchical relations of language and cognitive skills to comprehension of texts with other orthographies, such as Chinese, have not been examined. There were significant differences between Chinese, which has morphosyllabic orthography and alphabetic languages. One is that different from alphabetic languages, which have letter-to-phoneme correspondence, Chinese has character-to-syllable correspondence. The components of Chinese characters are arranged top to bottom, side by side, or inside-outside (Perfetti et al., 2013). These features of Chinese mean that one cannot sound out a character the same way they sound out a letter string in alphabetic languages through segmenting and blending processes. Another difference is that there is no inflectional system in Chinese, and thus the grammatical function of words is largely marked by word order, and the tense, degree, and number of words are decided by soliciting syntactic information from linguistic constituents (Li and Thompson, 1981; Chik et al., 2012a). Because of these differences, the weight of different skills (e.g., language skills) in predicting reading comprehension might vary across Chinese and alphabetic languages (Joshi et al., 2012; Cadime et al., 2017). Second, it is not known if comprehension monitoring longitudinally predicts reading, while other foundational language skills are included in the model (e.g., syntactic knowledge and orthographic knowledge in addition to vocabulary). The present study aimed to address these two critical gaps by investigating the direct and indirect effect of language skills and comprehension monitoring on reading Chinese, a language highly different from English, and by investigating the longitudinal relations of these skills to Chinese reading. Accordingly, this study has great potential for enhancing our theoretical understanding about the roles of language skills and comprehension monitoring in reading across languages as well as our understanding of effective practices in teaching reading.

Vocabulary is foundational to reading comprehension as readers need to decode meaning from words and phrases, which provides fundamental information for the building of basic propositions and the final mental representation of the text. Empirical research provides evidence for the concurrent relations of vocabulary to both listening comprehension and reading comprehension (Cromley and Azevedo, 2007; Kendeou et al., 2009; Tompkins et al., 2013; Kim, 2015). Among Chinese-speaking children, vocabulary was found to have a strong effect on not only word reading and word recognition (Tong et al., 2017) but also reading comprehension (Ku and Anderson, 2003; Chik et al., 2012b). Previous findings on the longitudinal relations between vocabulary and reading comprehension were inconsistent. For instance, in Zhang et al. (2013), Chinese children’s vocabulary at age 4 and 5 predicted their reading at age 9. In Li et al. (2012a), however, the vocabulary knowledge of Chinese children at age 8 did not predict their reading comprehension at age 10.

In English, semantic access to a word is usually done through phonological mediation, while, in Chinese, the mapping from orthography to semantics is more rapid than the mapping from orthography to phonology (Yang et al., 2006). Chinese is a morphosyllabic orthography, where a character corresponds to both a morpheme and a syllable (Shu et al., 2003). Since there is no grapheme-phoneme correspondence in Chinese, phonology cannot be activated incrementally with each grapheme-phoneme pair as it is done in alphabetic languages. In Chinese, phonology is not activated until the orthographic character is recognized as a whole unit. In Chinese, the orthographic system has three levels: stroke, radical, and character. Strokes (e.g., 丶) are combined to form radicals (e.g., 氵), which in turn are combined to form characters (e.g., 清). The majority of Chinese characters (80–90%) are semantic-phonetic, composing a semantic radical and a phonetic radical. The semantic radical provides information about the meaning of a character and the phonetic radical offers a clue to the pronunciation of the character. Most of the radicals have a legal position or rules within the character (Shu et al., 2003). For example, some radicals can only appear to the left (e.g., 亻), to the right (e.g., 刂), at the top (e.g., ⺮), or at the bottom (e.g., 灬). Therefore, orthographic knowledge in Chinese includes several aspects mainly the knowledge of structure, position, and function of semantic and phonetic radicals (Liu et al., 2017). Some studies show that orthographic knowledge is a significant predictor of Chinese word reading (e.g., Li et al., 2012b) and Chinese reading comprehension (e.g., Cheung et al., 2007). Longitudinal investigations on the role of orthographic knowledge in Chinese reading skills yielded inconsistent findings. For example, Yeung et al. (2012) found that the first grade orthographic knowledge predicted both second grade reading and fourth grade reading. Several other studies, however, show that orthographic skills did not predict children’s reading one year later (e.g., Tong et al., 2009; Chik et al., 2012a).

In addition to vocabulary knowledge and orthographic knowledge, syntactic knowledge is another important language skill needed for comprehension as readers need this information to group words into meaningful syntactic units, understand grammatical and semantic relations among words, and build coherence across sentences (Tong et al., 2014). Syntactic knowledge refers to one’s knowledge of the grammatical structure of a language (Gombert, 1992). Syntactic knowledge facilitates word recognition as readers can use syntactic constraints of a sentence to decode word meaning and make predictions about words that appear next in the sequence (So and Siegel, 1997). It aids sentence and text comprehension as readers can use this knowledge to integrate information at and above the sentence level (Tunmer and Hoover, 1992). Empirical research reveals that syntactic knowledge accounts for a significant amount of unique variance in concurrent sentence and text comprehension across different languages after controlling for variables such as vocabulary, working memory, and phonological awareness (Yeung et al., 2013; Tong et al., 2014). Longitudinal studies also indicate that syntactic knowledge remains a strong predictor of later reading comprehension after controlling for word-level reading skills (e.g., orthographic and morphological skills; Demont and Gombert, 1996; Chik et al., 2012a; Tong, 2013). Compared with alphabetic languages, such as English, syntax in Chinese includes a flexible word order, no inflectional system, and more extensive use of connectives (Li and Thompson, 1981). These syntactic features of Chinese make one’s syntactic knowledge especially important in recognizing Chinese words in context and extracting and integrating ideas at the sentence and text-level (Chik et al., 2012a).

Comprehension monitoring refers to a reader’s ability to evaluate and regulate his/her understanding of the text (Oakhill et al., 2003; Kim, 2015). To have successful comprehension, readers need to go beyond the representation of words and sentences and construct a coherent mental representation of the text, which involves the integration of initial and possibly incoherent propositions (Kintsch and Rawson, 2007; Perfetti et al., 2007). Accordingly, comprehension monitoring is necessary in the construction-integration process as readers need to constantly evaluate their understanding against the larger context or their world knowledge, detect inconsistencies or even contradictory information, and adopt proper strategies to solve understanding failures (Cain et al., 2004; Kendeou et al., 2009; Connor et al., 2015). Empirical evidence shows that comprehension monitoring explains unique variance in reading comprehension (Cain et al., 2004; Wong et al., 2017) across different languages. For instance, Wong et al. (2017) found that comprehension monitoring made a unique contribution to reading comprehension after controlling for age, nonverbal intelligence, word reading, and various aspects of oral language among Cantonese Chinese-speaking children. Very few studies have investigated the longitudinal contribution of comprehension monitoring to reading. In a recent study by LARRC and Yeomans-Maldonado (2017), comprehension monitoring at Grade 1 predicted English-speaking children’s reading comprehension at Grade 3 after controlling for vocabulary, decoding, and working memory.

Comprehension monitoring might mediate the relations between language skills and reading comprehension, based on the evidence that comprehension monitoring is a high-order cognitive skill built on more foundational language skills (Oakhill et al., 2003; Cain et al., 2004; Kim, 2015). That is, language skills (e.g., vocabulary and syntactic knowledge) may be represented as the underpinnings that support comprehension monitoring (Perfetti et al., 2007), which subsequently support reading comprehension. Empirical studies have supported the importance of language skills to comprehension monitoring. For instance, vocabulary knowledge is found to be a powerful predictor for comprehension monitoring (Cain et al., 2004; Perfetti et al., 2007). Meaning of related words, such as synonyms, antonyms, and category exemplars, in the text provides clues for internal consistency or inconsistency (Zargar et al., 2020). A rich semantic network of words associated with the topic makes it easier for readers to detect inconsistency between propositions built from the text and world knowledge (Currie and Cain, 2015). Syntactic knowledge helps children to monitor their comprehension more effectively (Oakhill et al., 2003). Better syntactic knowledge entails that children can better understand relations among words and sentences, and thus it is easier for them to detect inconsistencies. Children can use their syntactic knowledge to check their understanding against the larger grammatical context and better follow the content and structure, which facilitates comprehension monitoring (Tunmer and Hoover, 1992; Oakhill et al., 2003). Studies have also suggested that comprehension monitoring should be considered as a correlate and potential cause of individual differences in reading skills beyond language skills (Cain et al., 2004; Wong et al., 2017). Two recent studies conducted with children in Korea and the United States found that comprehension monitoring partially mediated the relations between language skills and listening comprehension (Kim, 2015, 2016). No studies that we are aware of, however, have tested the direct and mediated relations of language skills and comprehension monitoring to Chinese reading comprehension. Clearly, more studies in this line of research are needed.

Review of previous literature indicates that both language skills (i.e., vocabulary, orthographic knowledge, and syntactic knowledge) and comprehension monitoring make contributions to reading comprehension. Though the direct relations of these skills to reading comprehension are supported by previous research, the indirect relation of language skills *via* comprehension monitoring to reading comprehension has not been explored among Chinese-speaking children. Given the differences in orthography and syntactic rules between Chinese and English as noted above, it is worthwhile to see if findings on the mediation role of comprehension monitoring (Kim, 2015, 2016, 2020) can be generalized into Chinese. Few empirical studies have investigated the longitudinal relations of these skills to reading comprehension, and the findings were inconsistent (Chik et al., 2012a; Li et al., 2012a). Therefore, this study also aimed to examine the longitudinal associations of these skills to reading comprehension, another important yet less investigated area. The following two specific research questions guided the present study.

1. How do language skills in Grade 1 (i.e., vocabulary, syntactic knowledge, and orthographic knowledge) relate to comprehension monitoring in Grade 1? Does first grade comprehension monitoring partially or completely mediate the relations of language skills to reading comprehension in Grade 1?
2. Do language skills and comprehension monitoring in Grade 1 predict later reading comprehension in Grade 3?

With regard to the first research question, on the basis of existing research regarding the relations between language skills and comprehension monitoring (e.g., Oakhill et al., 2003; Perfetti et al., 2007; LARRC and Yeomans-Maldonado, 2017), we hypothesized that vocabulary, syntactic knowledge, and orthographic knowledge in Grade 1 would directly predict comprehension monitoring in Grade 1. Drawing from the construction-integration model (Kintsch and Rawson, 2007) and recent empirical studies (Kim, 2015, 2016), we tentatively hypothesized that comprehension monitoring in Grade 1 would partially mediate the relations between language skills and reading comprehension in Grade 1. With regard to the second research question, drawing from the existing research regarding the longitudinal associations between different component skills and reading (Saarnio et al., 1990; Cain et al., 2004; Vellutino et al., 2007; Garcia and Cain, 2014), we tentatively hypothesized that the word-level language skills (i.e., vocabulary and orthographic knowledge) in Grade 1 might not predict reading comprehension in Grade 3, but sentence-level language skill (i.e., syntactic knowledge) and higher-level cognitive skill (i.e., comprehension monitoring) might predict reading comprehension in Grade 3.

## Materials And Methods

### Participants

A total of 89 Chinese children (48 boys, *M_age_* = 86 months, *SD* = 4.09) were followed from Grade 1 to Grade 3 in this longitudinal study. The participants were from two intact classes in a public elementary school in an urban area in east China. A convenient sample was used; participants (i.e., children) were recruited through a teacher who volunteered to participate in the present study. There were six first-grade classes in this primary school, and the two classes taught by the teacher participated in the study. All students in these two classes participated. The participants were typically developing children, and they were monolingual Chinese speakers without any hearing or language impairments. The teacher who helped us with data collection advised that we should not collect information on annual income given due to its sensitivity. Based on the information given by the teacher, most of the families living in the school district are middle class families in China with an annual income of $20,000–40,000. The participants from the two classes are the representative of primary school students from urban middle class families in east China in terms of their age, gender, and reading developmental phase. In Grade 3, 82 students remained, with an attrition rate of 7.8%. The students dropped out of the study mainly due to transfer to other schools. Those who remained in the study and those who dropped out did not differ in skills measured in Grade 1. The study was conducted after obtaining consent from the parents of the participating children.

Reading is an important part of the Chinese curriculum in China, which is rather uniform in terms of the textbooks and instruction. In the Chinese classes, students used *Elementary Chinese Textbook* (2001), a popular Chinese textbook adopted by primary schools in many provinces in China. This textbook includes 12 volumes and students use one volume for each semester throughout the six primary school years. The Chinese class instruction usually focuses on reading the text aloud, the teaching of new Chinese characters in the text, sentence by sentence explanation, and the summary of main ideas.

### Measures

Children were assessed on their vocabulary, orthographic knowledge, syntactic knowledge, comprehension monitoring, and reading comprehension in Grade 1. Reading comprehension was assessed again in Grade 3. Children’s answers were dichotomously scored (correct = 1 and incorrect = 0) for each item unless otherwise noted.

#### Vocabulary

The Chinese version of The Peabody Picture Vocabulary Test-Revisited (PPVT-R, Dunn and Dunn, 1981; Sang and Miao, 1990) was used to measure children’s vocabulary. In this task, each word item had four picture illustrations. The children heard a word and were asked to select a picture that best corresponded to the word. The children were first given instructions and two trial items before they started the test items. They were told that if they were not sure about a word or did not know a word, they could make a guess. The task included 60 items with increasing difficulty. The reliability of PPVT-R in this study was 0.82.

#### Orthographic Knowledge

Children’s orthographic knowledge was assessed using a measure from Luo et al. (2011). This measure assessed the students’ awareness of internal structure of the Chinese character. Children were asked to judge which character looked more like a real Chinese character between a pair of pseudocharacter and noncharacter. This measure contained two practice items and 12 test items. In each item there was a pseudocharacter and a noncharacter. A pseudocharacter was constructed by replacing one radical of a real character with a position-legal radical, but it was not a real character (e.g., 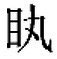). The noncharacter had its radicals in legal positions but one of the radicals had one stroke missing or added to it (e.g., 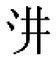). The reliability of this measure was 0.72 in this sample.

#### Syntactic Knowledge

Children’s syntactic knowledge was measured using the cloze task from So and Siegel (1997). This task assessed participants’ knowledge of basic syntactic structures in sentences. It included two practice items and 17 test items with one word missing in each sentence. The class of missing words included classifiers, nouns, verbs, prepositions, adjectives, adverbs, conjunctions, and particles. The children were first given the instructions that there was a missing word in each sentence and that the tester would say blank in place of the missing word. Children were asked to fill in a word in the blank that they thought would make the sentence sound right. They can write the word either in Chinese character or Pinyin. The children were given enough time to fill in each blank before the tester moved on to the next sentence. The test papers were scored according to the syntactic appropriateness of the responses in the sentence. The first author and her graduate student double coded answers from 25 participants. The inter-rater agreement was 98.6%. The reliability of this task in this sample was 0.80.

#### Comprehension Monitoring

An inconsistency detection test from Kim (2015) was used to assess children’s comprehension monitoring skill. In this test, children were asked to identify if a story made sense. The meaning of “not making sense” was explained to children during the practice items. If children answered that the story did not make sense, they were asked to provide a brief explanation. The test included two practice items and nine test items. Feedback and explanations were given in the practice items. An example of inconsistent item is as follows, “Xiaoli’s favorite color is blue. She wears blue every day. She has blue pants, blue shirts, and even blue shoes. Xiaoli likes to have everything purple!” An example of consistent item is as follows, “Xiaodan’s favorite thing to do is dance. She dances everywhere she goes. She dances at home. She dances at a park. Xiaodan even dances when she watches TV.” There were three consistent stories and six inconsistent stories in this task, randomly spread out across the items. For the six inconsistent stories, the accuracy of children’s explanations was also dichotomously scored. Therefore, the total possible score for this test was 15 (9 + 6). The reliability of this measure was 0.65.

#### Reading Comprehension

Children’s reading comprehension in Grade 1 was assessed using four reading passages (level 1) adapted from *Comprehensive Reading Inventory* (*CRI*, Cooter et al., 2006). The passages were translated from English to Chinese by the first author who had extensive experience in translation. The words were carefully chosen to ensure that the passages were at an appropriate difficulty level for the children. The passage length was similar to that of texts in their textbooks, varying between 134 and 174 Chinese characters. We asked three primary teachers teaching Grade 1 Chinese read the passages, and all of them deemed the difficult level and question format as developmentally appropriate for the first graders at the end of spring semester. In this task, children were asked to read the passages in silence and then the tester read the questions following each passage. The texts were left with children for consulting when they answered the questions. There were two types of questions: literal and intratextual inference. All questions from the original measure CRI were retained, but the format of some questions (three out of eight) was altered from the short answer to multiple questions as a combination of these two question formats usually appear in reading comprehension tasks for Chinese children. For the short answer questions, children can write down the answer either in Chinese characters or Pinyin. One point was given for each correct answer. The first author and her graduate student double coded answers from 25 participants. The inter-rater agreement for the four reading passages was 98.5, 98.5, 99, and 99%, respectively. The total possible score for each reading passage is 8. The reliability for the four reading passages was 0.67, 0.68, 0.69, and 0.72.

Children’s reading comprehension in Grade 3 was assessed using two reading passages (level 3) adapted from *Comprehensive Reading Inventory* (CRI; Cooter et al., 2006) and one reading passage adapted from *Progress in International Reading Literacy Study* (PIRLS, 2006). The two reading passages from CRI were translated from English to Chinese by the first author. All questions from the original measure CRI were retained, but the format of some questions (three out of eight) was changed from short answer to multiple choice format. Two types of questions were focused on, i.e., literal and intratextual inference. The PIRLS reading test was an international test targeting at 9-year old children. It was administered every 5 years to monitor and compare the reading performance of children from different countries. The original passage from PIRLS was followed by 13 questions in either multiple choice or short answer format. We retained 11 of the questions considering the time constraint and kept the original question format. Children read the three passages and answered the questions in silence. One point was given for each correct answer. The first author and her graduate student double coded answers from 25 participants. The inter-rater agreement was 98.5, 99, 98.5% for the three reading passages. The total possible score for each passage was 8, 8, and 11. The reliability for the three reading passages was 0.62, 0.72, and 0.68.

### Procedures

The tasks were administered to the children in several different sessions. The comprehension monitoring measure was administered to children individually in a quiet room at school by a rigorously trained graduate student. This individual session lasted about 15 min for each child. All the other measures were administered by a trained primary school teacher in group sessions. The duration for each group session was around 35 min. The teacher received a 2-h face-to-face training from the researcher before administering the tasks. The training was composed of two parts. In the first part, the teacher was given a sheet of detailed instructions for each task and the researcher explained to her the procedures involved in each task. In the second part, she watched the researcher model administering all the tasks and then completed a mock administration of all tasks herself with the researcher, during which she received feedback regarding the administration. At the end of each group session, the teacher collected the task papers and gave them to the researcher, who then scored the papers with a trained graduate student.

### Data Analysis Strategy

**Figure 1:**
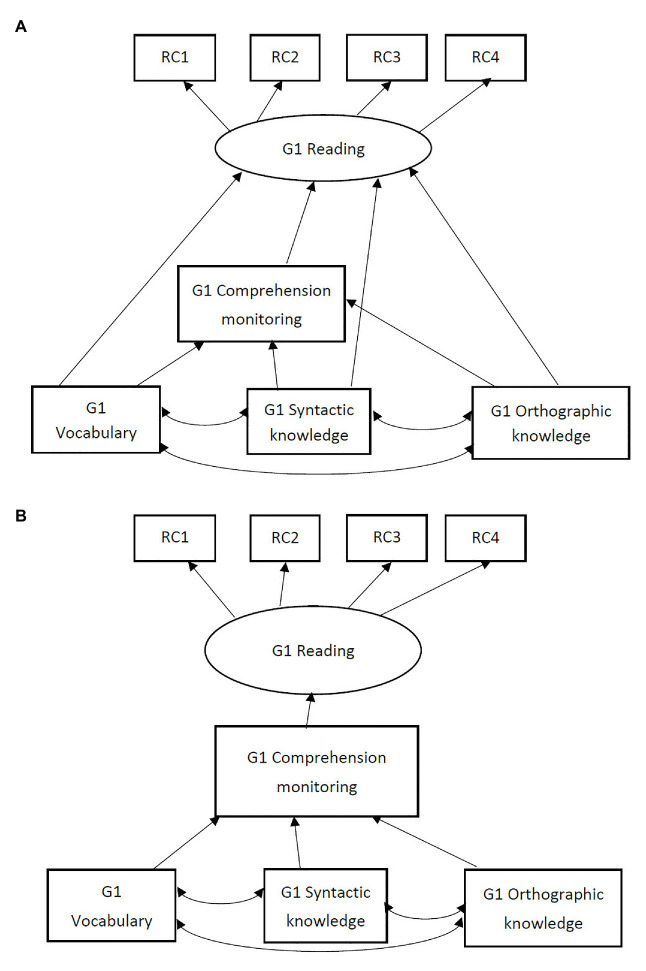
Two alternative models of concurrent relations among Grade 1 reading, comprehension monitoring, and language skills. (A) Partial mediation model; (B) complete mediation model. GI = Grade 1; RC 1/2/3/4 = Reading comprehension task 1/2/3/4 in Grade 1.

**Figure 2:**
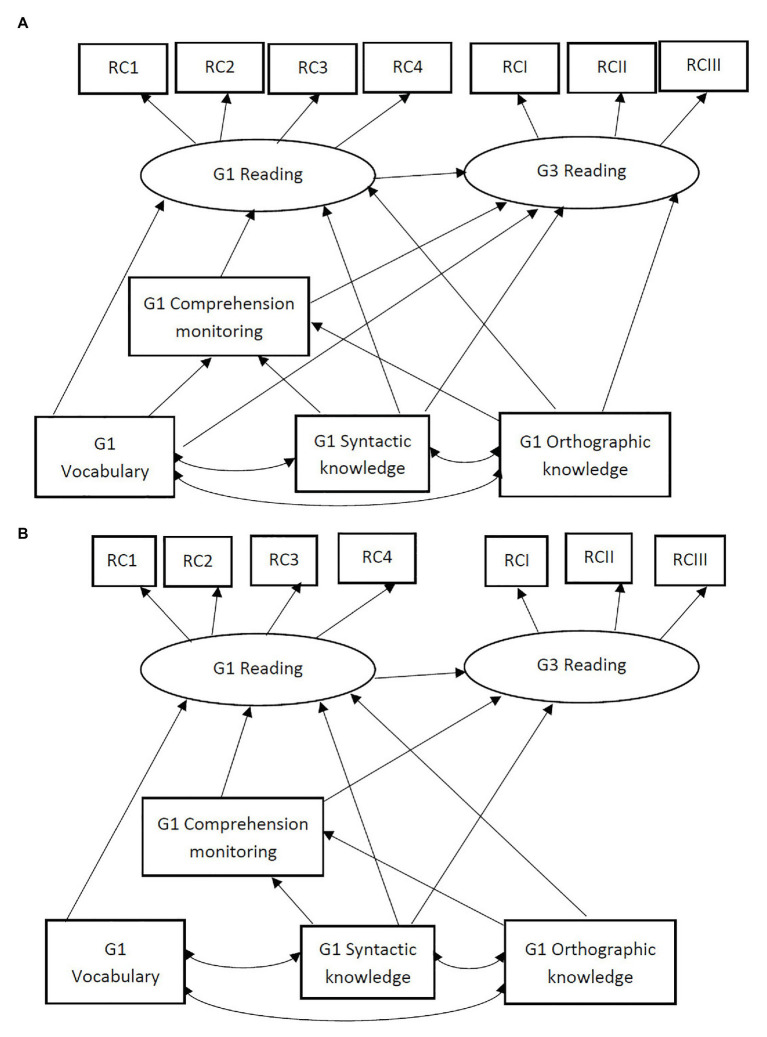
Two alternative models of longitudinal relations among Grade 3 reading, Grade 1 reading, comprehension monitoring, and language skills. In (A), vocabulary and orthographic knowledge in Grade 1 are directly and indirectly related to Grade 3 reading; In (B), vocabulary and orthographic knowledge in Grade 1 are indirectly related to Grade 3 reading. G1 = Grade 1; G3 = Grade 3; RC 1/2/3/4 = Reading comprehension task 1/2/3/4 in Grade 1; RCI/II/III = Reading comprehension task 1/2/3 in Grade 3.

The primary data analysis strategy was a structural equation model (SEM), using the R package lavaan (Rosseel, 2012). Grade 1 reading comprehension and Grade 3 reading comprehension were specified as latent outcome variables indicated by four reading passages and three reading passages, respectively. Other constructs were assessed by single measures and thus observed variables were used. Research question one about the concurrent relations among language skills, comprehension monitoring, and reading comprehension was addressed by fitting and comparing the two alternative models in Figure 1 using data from Grade 1. Figure 1A presents the partial mediation model, in which language skills (vocabulary, syntactic knowledge, and orthographic knowledge) were hypothesized to have both direct effects on Grade 1 reading comprehension, and indirect effects *via* comprehension monitoring. Figure 1B presents the complete mediation model, in which language skills were hypothesized to have only indirect effects on Grade 1 reading comprehension *via* comprehension monitoring. Research question two about the longitudinal relations was addressed by fitting and comparing two alternative models in Figure 2 using data from Grade 1 and Grade 3. In Figure 2A, all the component skills in Grade 1 were hypothesized to have direct contributions to Grade 3 reading comprehension in addition to Grade 1 reading comprehension. In Figure 2B, syntactic knowledge and comprehension monitoring were hypothesized to have direct contributions to Grade 3 reading comprehension in addition to Grade 1 reading comprehension while vocabulary and orthographic knowledge do not.

For Grade 1 data, there were two observations with missing data on two variables and three observations with missing data on one variable. For Grade 3 data, there were three observations with missing data on one variable and two observations with missing data on two variables. Missing data were found to be missing completely at random (Little’s MCAR test *χ*^2^ = 108.24, *df* = 87, *p* = 0.57) and were treated using the full information maximum likelihood method (Enders, 2010). Model fit was evaluated by the *χ*^2^ statistic, root mean square error of approximation (RMSEA), comparative fit index (CFI), Tucker-Lewis index (TLI), and Standardized Root Mean Square Residual (SRMR). Good model fit was indicated by CFI above 0.95, RMSEA below 0.05, and SRMR below 0.08 (Browne and Cudeck, 1993; Hu and Bentler, 1999; Brown, 2006). Model comparisons were made using Chi square differences. Each indirect effect was deemed statistically significant if the 95% bias-corrected bootstrap confidence intervals (CIs) did not contain zero (Preacher and Hayes, 2004).

## Results

The descriptive statistics and intercorrelations of the variables in this study are presented in Table 1. Vocabulary, orthographic knowledge, and comprehension monitoring were weakly to moderately related to Grade 1 and Grade 3 reading comprehension (0.08 ≤ *r* ≤ 0.47). Syntactic knowledge was moderately to strongly related to Grade 1 and Grade 3 reading comprehension (0.42 ≤ *r* ≤ 0.62). Vocabulary, orthographic knowledge, and syntactic knowledge were moderately related to each other (0.34 ≤ *r* ≤ 0.41). The three language skills were weakly to moderately related to comprehension monitoring (0.23 ≤ *r* ≤ 0.45). Grade 1 reading comprehension was weakly to moderately related to Grade 3 reading comprehension (0.27 ≤ *r* ≤ 0.46).

**Table 1:** Descriptive statistics and bivariate correlations among study variables.

	(1)	(2)	(3)	(4)	(5)	(6)	(7)	(8)	(9)	(10)	(11)
(1) Vocabulary	-										
(2) Syntactic knowledge	0.34^**^	-									
(3) Orthographic knowledge	0.41^**^	0.39^**^	-								
(4) Comprehension monitoring	0.45^**^	0.41^**^	0.23^*^	-							
(5) G1 reading comprehension 1	0.22^*^	0.44^**^	0.08	0.22^*^	-						
(6) G1 reading comprehension 2	0.32^**^	0.44^**^	0.17	0.47^**^	0.44^**^	-					
(7) G1 reading comprehension 3	0.33^**^	0.53^**^	0.28^**^	0.38^**^	0.49^**^	0.63^**^	-				
(8) G1 reading comprehension 4	0.36^**^	0.42^**^	0.27^*^	0.23^*^	0.27^**^	0.42^**^	0.44^**^	-			
(9) G3 reading comprehension 1	0.29^*^	0.62^**^	0.25^*^	0.27^*^	0.46^**^	0.36^**^	0.44^**^	0.38^**^	-		
(10) G3 reading comprehension 2	0.31^**^	0.62^**^	0.42^**^	0.38^**^	0.36^**^	0.33^**^	0.37^**^	0.27^*^	0.70^**^	-	
(11) G3 reading comprehension 3	0.40^**^	0.44^**^	0.41^**^	0.33^**^	0.29^*^	0.28^*^	0.33^**^	0.33^**^	0.43^**^	0.48^**^	-
*Min*	27	1	0	4	3	1	1	2	0	0	1
*Max*	57	17	12	15	8	8	8	8	8	8	10
*M*	47.30	12.39	9.20	9.86	6.98	6.13	6.17	6.63	5.56	5.99	6.28
*SD*	6.12	3.01	2.32	2.39	1.07	1.60	1.58	1.46	1.70	1.92	2.18

G1 = Grade 1 and G3 = Grade 3.

* *p* < 0.05

; ** *p* < 0.01

**Figure 3:**
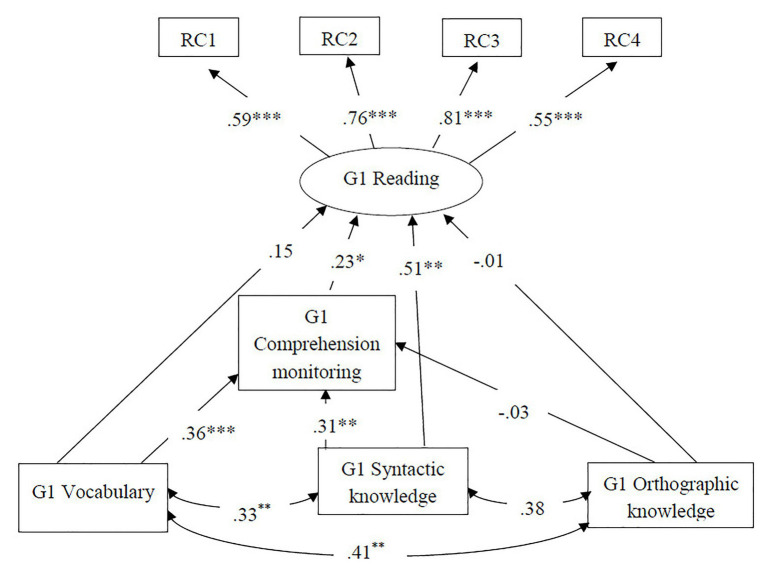
Concurrent relations among language skills, comprehension monitoring, and reading comprehension in Grade 1. Standardized estimates were reported. G1 = Grade 1; RC 1/2/3/4 = Reading comprehension task 1/2/3/4 in Grade 1; ^*^p < 0.05, ^**^p < 0.01, and ^***^p < 0.001.

In order to examine the concurrent relations of language skills and comprehension monitoring to Grade 1 reading comprehension, the two alternative models in Figure 1 were fitted to the data. The partial mediation model (Figure 1A) had an excellent model fit, *χ*^2^ = 14.69, *df* = 14, *p* = 0.400, CFI = 1.00, TLI = 0.99, RMSEA = 0.02, SRMR = 0.04. The complete mediation model (Figure 1B) had a poor model fit, *χ*^2^ = 39.19, *df* = 17, *p* = 0.002, CFI = 0.88, TLI = 0.81, RMSEA = 0.12, SRMR = 0.11. Chi square difference test showed that the partial mediation model was superior, Δχ^2^ = 24.50, Δ*df* = 3, *p* = 0.000. Standardized estimates are reported in Figure 3. Among the three language skills, vocabulary (*β =* 0.36, *p* = 0.000) and syntactic knowledge (*β* = 0.31, *p* = 0.003) were found to be a statistically significant predictors of comprehension monitoring, whereas orthographic knowledge did not predict comprehension monitoring (*β* = −0.03, *p* = 0.078). Syntactic knowledge (*β* = 0.51, *p* = 0.000) was significantly related to Grade 1 reading comprehension while vocabulary (*β* = 0.15, *p* = 0.231) and orthographic knowledge (*β* = −0.01, *p* = 0.915) were not significantly related to Grade 1 reading. After controlling for the three language skills, comprehension monitoring was a statistically significant predictor of Grade 1 reading comprehension (*β* = 0.23, *p* = 0.045). About 49.2% of variance of Grade 1 reading comprehension and 27.7% of variance of comprehension monitoring were explained by the predictors included in the model.

Tests of indirect effects revealed that comprehension monitoring played different roles in the relations between language skills and Grade 1 reading comprehension. The direct effect of vocabulary on reading comprehension in Grade 1 was not statistically significant (*c1* = 0.15, *SE* = 0.02, *p* = 0.340), whereas the indirect effect of vocabulary on reading comprehension in Grade 1 through comprehension monitoring was statistically significant (*a1b* = 0.08, 95% bootstrap CI: 0.002–0.023). Therefore, comprehension monitoring fully mediated the relation between vocabulary and Grade 1 reading comprehension. On the other hand, comprehension monitoring partially mediated the relation between syntactic knowledge and Grade 1 reading comprehension, as indicated by statistically significant direct effect (*c2* = 0.51, *SE* = 0.04, *p* = 0.003) and indirect effect (*a2b* = 0.07, 95% bootstrap CI: 0.003–0.040). Finally, neither the direct effect (*c3* = −0.01, *SE* = 0.04, *p* = 0.93) nor the indirect effect (*a3b* = −0.008, 95% bootstrap CI: −0.021–0.009) of orthographic knowledge was statistically significant, suggesting that comprehension monitoring did not mediate the relation between orthographic knowledge on Grade 1 reading comprehension. Table 2 shows the direct, indirect, and total effects of the included predictors on Grade 1 reading comprehension. The total effects of variables on Grade 1 reading comprehension were 0.23 for vocabulary, 0.58 for syntactic knowledge, −0.02 for orthographic knowledge, and 0.23 for comprehension monitoring.

**Table 2:** Direct, indirect, and total effect estimates of the model predictors on Grade 1 reading comprehension.

	Direct	Indirect	Total
Vocabulary	0.15 (0.02)	0.08 (0.005)^a^	0.23 (0.016)
Syntactic knowledge	0.51 (0.04)	0.07(0.008)^a^	0.58 (0.033)
Orthographic knowledge	−0.01 (0.04)	−0.01 (0.007)	−0.02 (0.037)
Comprehension monitoring^b^	0.23 (0.03)	--	--

a The indirect effects were statistically significant with bootstrapping.

b Comprehension monitoring only had a direct effect in the model.

The values reported are the standardized coefficients and the corresponding standard errors are in parentheses.

**Figure 4:**
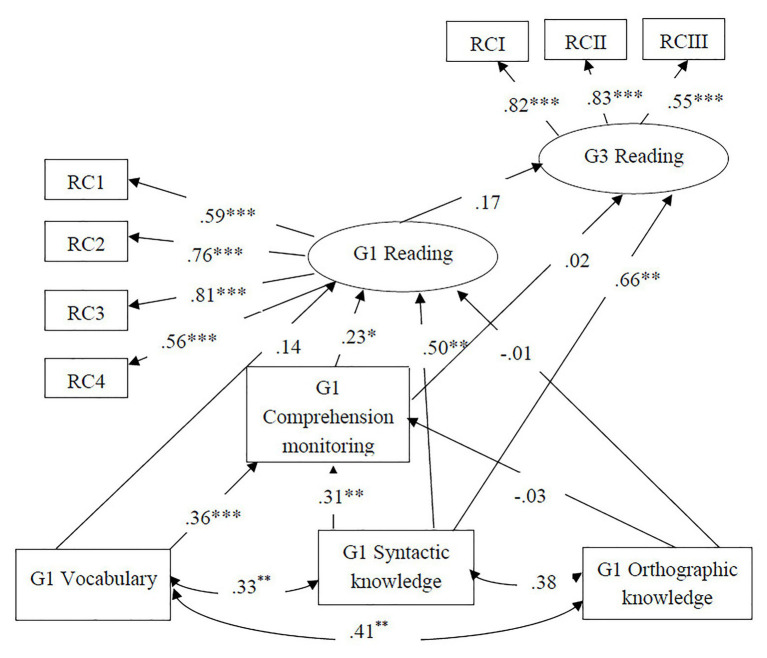
Longitudinal relations among language skills, comprehension monitoring, and reading comprehension in Grade 3. Standardized estimates were reported. G1 = Grade 1; G3 = Grade 3; RC 1/2/3/4 = Reading comprehension task 1/2/3/4 in Grade 1; RCI/II/III = Reading comprehension task 1/2/3 in Grade 3; ^*^p < 0.05, ^**^p < 0.01, and ^***^p < 0.001.

In order to examine the longitudinal relations of Grade 1 language skills and comprehension monitoring to Grade 3 reading comprehension, the two alternative models in Figure 2 were fitted to the data. The first model, Figure 2A, hypothesized that all the component skills in Grade 1 made direct contribution to Grade 3 reading comprehension in addition to their contribution to Grade 1 reading comprehension. This model had a good model fit, *χ*^2^ = 39.13, *df* = 33, *p* = 0.214, CFI = 0.98, TLI = 0.97, RMSEA = 0.05, SRMR = 0.06. The second model, Figure 2B, hypothesized that syntactic knowledge and comprehension monitoring made direct contribution to Grade 3 reading while vocabulary and orthographic knowledge did not. This model also had a good model fit, *χ*^2^ = 42.17, *df* = 35, *p* = 0.189, CFI = 0.98, TLI = 0.96, RMSEA = 0.05, SRMR = 0.07. Chi square difference test showed that there was no significant difference between the two models, Δ*χ*^2^ = 3.03, Δ*df* = 2, *p* = 0.220. The second model (Figure 2B) was chosen for parsimony. Standardized path coefficients are presented in Figure 4. Syntactic knowledge in Grade 1 significantly predicted Grade 3 reading comprehension (*β* = 0.66, *p* = 0.000) while comprehension monitoring (*β* = 0.02, *p* = 0.882) was not related to Grade 3 reading comprehension after accounting for its contribution to Grade 1 reading comprehension. Grade 1 reading (*β* = 0.17, *p* = 0.307) was not related to Grade 3 reading after accounting for the contributions of Grade 1 component skills. To check if our sample size is adequate to achieve desired statistical power, we run a power analysis for each structural path using Monte Carlo simulations. As is shown in Table 3, the statistical power of most significant structural paths reached the high power level ranging from 0.85 to 1.00 and one path (from comprehension monitoring to Grade 1 reading) reached medium power level (0.73) according to the cutoff of power level (≥0.80 for high power, 0.60–0.79 for medium power) recommended by Riedl et al. (2014). About 62% of variance in Grade 3 reading was explained by predictors included in the model. None of the indirect effects in the model were statistically significant, suggesting that comprehension monitoring did not mediate the longitudinal relations between Grade 1 language skills and Grade 3 reading comprehension. The total effects of variables on Grade 3 reading were 0.04 for vocabulary, 0.76 for syntactic knowledge, 0.00 for orthographic knowledge, 0.06 for comprehension monitoring, and 0.17 for Grade 1 reading comprehension.

**Table 3:** Direct effects of model predictors in the longitudinal model.

	Path coefficient	Standard errors	*t*	*p*	Statistical power
G1 reading → G3 reading	0.170	0.373	1.021	0.307	0.470
CM → G3 reading	0.017	0.066	0.149	0.882	0.066
SYN → G3 reading	0.660	0.076	4.074	0.000	1.000
CM → G1 reading	0.227	0.030	2.004	0.045	0.727
VOC → G1 reading	0.143	0.013	1.170	0.242	0.375
SYN → G1 reading	0.504	0.029	3.642	0.000	1.000
ORT → G1 reading	−0.004	0.029	−0.036	0.971	0.067
VOC → CM	0.358	0.040	3.507	0.000	0.936
SYN → CM	0.303	0.081	2.964	0.003	0.852
ORT → CM	−0.033	0.109	−0.308	0.758	0.063

G1 = Grade 1; G3 = Grade 3; CM = Comprehension monitoring; SYN = Syntactic knowledge; ORT = Orthographic knowledge.

## Discussion

The primary goal of this study was to investigate various language skills (vocabulary, syntactic knowledge, and orthographic knowledge) and comprehension monitoring and their direct and mediated relations to Chinese reading comprehension among young Chinese-speaking children from a developmental perspective. Specifically, we examined the relations of language skills and comprehension monitoring to concurrent reading comprehension in Grade 1 children as well as the relations of Grade 1 language skills and comprehension monitoring to reading comprehension in Grade 3. The results of the study highlight the direct and mediated nature of relations among these skills and longitudinal predictive relations of these skills to reading comprehension in the population of Chinese-speaking children, thus supporting the hierarchical and dynamic relations among componential skills of reading (Cromley and Azevedo, 2007; Kim, 2015, 2016, 2020). The major findings will be discussed, followed by the educational implications and limitations of the results and suggestions for future research directions.

### Concurrent Relations

Our findings indicate that Grade 1 comprehension monitoring completely mediates the relation between Grade 1 vocabulary and Grade 1 reading comprehension. More specifically, we found that vocabulary was directly related to comprehension monitoring after accounting for syntactic knowledge and orthographic knowledge and indirectly contributed to reading comprehension *via* comprehension monitoring. However, the direct relation of Grade 1 vocabulary to Grade 1 reading comprehension was not statistically significant after accounting for the other variables in the model. This finding is consistent with the evidence that vocabulary is a foundational language skill that supports comprehension monitoring in readers of alphabetic languages (Cain et al., 2004; Perfetti et al., 2007; LARRC and Yeomans-Maldonado, 2017). To extend this line of research, our finding indicates that vocabulary is necessary for comprehension monitoring in Grade 1 Chinese children.

This finding is discrepant from other research studies showing a direct relation between vocabulary and reading comprehension among both English-speaking and Chinese-speaking children (Cain et al., 2004; Cromley and Azevedo, 2007; Chik et al., 2012b). Inconsistent results may reflect key differences between our study and the previous studies and unique features of Chinese (e.g., morphosyllabic orthography). Scholars suggest that reading comprehension is linked to the semantic side or the quality of lexical representation (Perfetti, 1999; Roth et al., 2002; Ouellette, 2006). In Chik et al. (2012b), the oral vocabulary task asked students to make sentences with a list of printed words. This task taps both the children’s ability to derive meaning from printed words (from orthography to semantics), and their ability to use words in meaningful ways. This requires high quality of lexical representation including word class, collocation, semantic category, the knowledge of which can provide semantic cues in reading and facilitate the understanding of individual words and adjacent words (Oakhill, 2020). In our study, vocabulary knowledge was measured by PPVT, which assessed children’s breadth of vocabulary knowledge with a listening task. Since Chinese is a morphosyllabic orthography with no phoneme-grapheme correspondence, understanding a word in listening (from phonology to semantics) does not mean that one can readily understand it in reading (from orthography to semantics), because he cannot sound out a character the way he sounds out an alphabetic word. It is very natural that Chinese children might understand a word in listening but cannot derive its meaning in printed form (Li et al., 2012a). Therefore, a large listening vocabulary size might not aid Chinese reading comprehension as much as it aids reading in an alphabetic language (Cain et al., 2004). Another potential explanation may be that these previous studies (Ku and Anderson, 2003; Chik et al., 2012b) did not include higher-level cognitive skills such as comprehension monitoring. Our finding indicates that much of the contribution of vocabulary to reading comprehension is *via* comprehension monitoring.

The significant indirect effect from vocabulary to reading comprehension *via* comprehension monitoring supports the construction-integration model (Kintsch, 1988; Kintsch and Rawson, 2007; Perfetti et al., 2007) and extends previous research, supporting the indirect relations between vocabulary and listening comprehension (Kim, 2015, 2016). The finding indicates that children utilize their vocabulary knowledge to help them to connect propositions in the text and detect inconsistencies in construction, and to monitor their level of comprehension, which in turn improves their reading comprehension. Taken together, these findings support that vocabulary is critical for reading comprehension, contributing indirectly *via* a higher-order cognitive skill (i.e., comprehension monitoring) for Chinese children in Grade 1.

Our finding confirms our hypothesis that comprehension monitoring partially mediates the relation between syntactic knowledge and reading comprehension in Grade 1. More specifically, syntactic knowledge was directly associated with comprehension monitoring when controlling for the contributions of vocabulary and orthographic knowledge and indirectly contributed to reading comprehension through comprehension monitoring. This result is in line with previous work supporting the role of syntactic knowledge in comprehension monitoring (Tunmer and Hoover, 1992; Oakhill et al., 2003) and extends previous studies (Kim, 2015, 2016) by confirming the mediating role of comprehension monitoring between syntactic knowledge and reading comprehension. Syntactic knowledge was also directly related to reading comprehension, which is consistent with previous findings that syntactic knowledge is important to reading comprehension (Chik et al., 2012a; Tong et al., 2014). These results indicate that syntactic knowledge is a foundational skill for constructing propositions needed not only for comprehension monitoring but also building the situation model at the discourse level for first-grade Chinese children.

In contrast to vocabulary and syntactic knowledge, however, the direct relation of orthographic knowledge to reading comprehension and its indirect relation to reading comprehension in Grade 1 *via* comprehension monitoring were not significant. One potential explanation for nonsignificant relations of orthographic knowledge to reading comprehension may be that vocabulary and syntactic knowledge are more powerful predictors, or that orthographic knowledge, vocabulary knowledge, and syntactic knowledge are interdependent. Our finding is contradictory to Cheung et al. (2007), which found significant relations between orthographic knowledge and text comprehension in Chinese children. Contradictory findings may be caused by the differences in the methods of measuring orthographic knowledge. Cheung et al. (2007) examined children’s knowledge about both the position and function of Chinese radicals, while our measures of orthographic knowledge mainly assessed children’s knowledge of the position of radicals. It may be that reading comprehension is more closely related to children’s knowledge about the function of radicals than the position of radicals as children can use their knowledge of function of radicals to attend to phonological and semantic cues of radicals to extract sound and meaning of Chinese characters, which facilitates text comprehension (Cheung et al., 2007).

In regards to comprehension monitoring, we found that this cognitive skill measured at Grade 1 was significantly related to reading comprehension, supporting the hypothesis that initial propositions need to be evaluated and integrated with other propositions to build coherence in their text representation (Perfetti et al., 2007). The current finding is in line with recent evidence that comprehension monitoring is important to reading comprehension (Cain et al., 2004; Wong et al., 2017). Our result extends the finding of Kim (2015) with a different population, demonstrating that the mediated relations between the lower-level skills of vocabulary and syntax and reading comprehension *via* the higher-level skill of comprehension monitoring generalize across languages.

### Longitudinal Relations

It is notable that syntactic knowledge in Grade 1 in terms of word order, connective usage, and knowledge of morphosyntactic structure stood out as the only variable that longitudinally predicted reading comprehension in Grade 3, after accounting for the other skills in Grade 1. This finding is convergent with previous studies showing that syntactic knowledge remains a significant predictor for reading comprehension from beginning to advanced phase of reading development (Chen et al., 1993; Chik et al., 2012a; Tong, 2013). The syntactic knowledge that children acquired in Grade 1, including morphosyntactic structure, connectives, and word order, is still much needed in comprehending Grade 3 reading passages, which are more challenging to read by containing more complex sentence structures and longer sentences (Tsang and Stokes, 2001; Chik et al., 2012a,b).

The strong predictive power of syntactic knowledge to concurrent and later Chinese reading comprehension may be explained by the special features of Chinese syntax. In Chinese reading, syntactic knowledge is especially important for reading comprehension, because there is no inflectional system, more flexible word order, and more extensive use of connectives (Li and Thompson, 1981). First, since there is no inflectional system, such as subject-verb agreement or case marking in Chinese, instead of the morphological transformations, readers need to solicit syntactic information from linguistic constituents and their semantic relationships to decide the tense, number, degree and word class. Syntactic knowledge can provide the context in which a word is interpreted and thus is vital to comprehension at the sentence level (Bishop and Snowling, 2004). Second, since there are no morphological transformations, the grammatical function of words are largely marked by their order in the sentence. Word order thus plays a significant role in understanding the meaning of individual words and the meaning embedded in specific sentence structures (Chik et al., 2012a). Compared with English, Chinese has a more flexible word order. Besides the regular subject-verb-object order, topic-comment sentence structure is widely used (Li and Thompson, 1981). Once a topic is established, it can be extended to succeeding sentences, and subjects of these sentences can be omitted. Therefore, a good mastery of word order not only facilitates building of meaning within sentences but also the integration of meaning across sentences. Third, connectives or conjunction words denoting time, reason, contrast, condition, and progressive relations are widely used in Chinese as a cohesive device. The understanding of connectives helps one to tell the logical relations between clauses, better follow semantic traces between sentences, and establish text coherence, which is essential to successful text comprehension (Koda, 2005).

As we hypothesized, vocabulary knowledge and orthographic knowledge measured in Grade 1 did not contribute to Grade-3 reading comprehension. These results are consistent with the previous longitudinal studies, which showed no predictive power of vocabulary and orthographic knowledge on later reading comprehension (e.g., Chik et al., 2012a; Li et al., 2012a). These results are discrepant from a longitudinal prediction of vocabulary knowledge tested at ages 4 and 5 years old to the third grade reading (Zhang et al., 2013) and a longitudinal prediction of the first grade orthographic knowledge to the second grade reading and fourth grade reading (Yeung et al., 2012). However, direct comparison of these results requires caution as studies differ in how vocabulary, orthographic knowledge, and reading were measured in these studies. For example, both Yeung et al. (2012) and Zhang et al. (2013) used the measures of Chinese word reading to indicate children’s Chinese reading skills. By contrast, in Chik et al. (2012a) and Li et al. (2012a), and our study, reading was measured at the sentence or text level. Therefore, one way to interpret the present findings is that vocabulary knowledge and orthographic knowledge loses their unique and independent predictive power to later sentence-level and text-level reading comprehension, but they are still important predictors of later word reading skills.

Contrary to our hypothesis, Grade-1 comprehension monitoring was not related to Grade 3 reading comprehension. This finding is inconsistent with LARRC and Yeomans-Maldonado (2017), which demonstrated that the first grade comprehension monitoring predicted the third grade reading comprehension. These inconsistent findings might be due to the fact that different variables were included in the models. In LARRC and Yeomans-Maldonado, working memory, vocabulary, word decoding, and comprehension monitoring were included as predictors for reading, while in our model, syntactic knowledge, vocabulary, orthographic knowledge, and comprehension monitoring were included. It might be that the foundational language skills especially syntactic knowledge in our model shared some of the variances in comprehension monitoring and reduced the longitudinal predictive power of comprehension monitoring. To check this explanation, we excluded syntactic knowledge from the longitudinal model and rerun the analysis. Results showed that Grade 1 comprehension monitoring made indirect contribution to Grade 3 reading comprehension through Grade 1 reading comprehension (*a1b1* = 0.18, 95% bootstrap CI: 0.02–0.21). This result confirmed that the difference in our study, and LARRC and Yeomans-Maldonado is mainly due to the different variables included in the models. Therefore, we tentatively suggest that both Grade 1 comprehension monitoring and syntactic knowledge are important early predictors to Grade 3 reading comprehension, but syntactic knowledge tends to override the importance of comprehension monitoring.

In summary, the language skills (vocabulary, syntactic knowledge, and orthographic knowledge) and comprehension monitoring measured in Grade 1 explained 49.2% of the variance in Grade-1 reading comprehension skills among a sample of Chinese-speaking children. This finding is similar to the studies with Italian-speaking children showing that 44–54% of variance in listening comprehension was explained by language (e.g., vocabulary) and cognitive skills (e.g., inferential skills; Florit et al., 2014). In comparison, Kim’s studies (Kim, 2015, 2016) explained a larger amount of variance in listening comprehension (74–86%) and reading comprehension (66%). This may be caused by more predictors such as working memory and the theory of mind included in the studies conducted by Kim (2015, 2016). Importantly, we examined both the direct and indirect effects of language skills and comprehension monitoring on reading comprehension in Chinese-speaking children. Indirect effects made differences in accounting for the total effects of vocabulary and syntactic knowledge on reading comprehension, thereby highlighting the importance of taking both direct and indirect effects into consideration. When Grade 3 reading comprehension was included, the model explained 62% of variance in Grade 3 reading comprehension. Grade-1 syntactic knowledge had a substantial – and the largest – direct effect on Grade-3 reading comprehension (0.76), suggesting that syntactic knowledge is one of the important foundation skills that underpin Chinese reading comprehension at the text level.

## Implications

This study confirmed that multiple language skills and comprehension monitoring are involved in comprehension at the text level and these skills are directly and indirectly involved in reading comprehension for Chinese-speaking children. These findings have important implications, albeit preliminary due to the correlational nature of the current study. First, the unique contributions of syntactic knowledge and comprehension monitoring to reading comprehension suggest that it is imperative for Chinese teachers to attend to these languages and cognitive skills in instruction. Second, the significant indirect relation of vocabulary knowledge to reading comprehension *via* comprehension monitoring implies that vocabulary skills help children to improve their awareness about their level of comprehension which in turn enhances their reading comprehension. Therefore, this finding suggests that reading instruction might target vocabulary and comprehension monitoring simultaneously for Chinese children who are still in the “learning to read” phase. Third, both the direct relation of syntactic knowledge to reading comprehension and the indirect relation of syntactic knowledge to reading comprehension *via* comprehension monitoring were significant. This finding suggests that Chinese children might benefit from a combination of teaching aimed at improving reading, with the promotion of syntactic knowledge and comprehension monitoring. Furthermore, the longitudinal predictive power of syntactic knowledge underscore the importance of developing this skill for future reading comprehension ability.

## Limitation and Future Directions

Despite the important implications of this study, some limitations warrant note. First, because this is a correlational study, we cannot assume that any significant relationship obtained from our study is causal in nature. Second, although important language and cognitive predictors were included in the study, some potential predictors of reading comprehension were not examined. Those predictors include phonological knowledge (Pan et al., 2016), morphological knowledge (Zhang et al., 2012), inference making (Tompkins et al., 2013), working memory (Spencer et al., 2019), the theory of mind (Kim, 2015, 2016), and text structure knowledge (Cain et al., 2004). For example, working memory, as a basic cognitive skill maintaining and processing information, is important to reading comprehension as there is frequent integration of information across sentences (Oakhill, 2020). Future studies should include these predictors to expand our understandings about important reading skills that may influence Chinese reading comprehension development. Third, we focused on the impact of Grade 1 component skills on Grade 3 reading comprehension. Without Grade 3 predictors, we cannot compare the contribution of component skills to reading comprehension across different grade levels. In future studies, it would be also interesting to examine both early predictors (the first grade variables) and concurrent predictors (the third grade variables). Fourth, though the comprehension monitoring measure adopted in our study is a widely used measure (Cain and Oakhill, 2006; Kim, 2016, 2020; LARRC and Yeomans-Maldonado, 2017), it mainly focuses on the internal inconsistency of the text. As comprehension monitoring also copes with lexical difficulties, syntactic acceptability, internal consistency, and external consistency, future studies may adopt a measure that taps all these aspects, which might yield a more comprehensive representation of the children’s comprehension monitoring ability. Fifth, due to time and resource constraints, the study had a relative small sample size and single measures were used to assess vocabulary, syntactic knowledge, and comprehension monitoring. As such, latent variables were not created for these languages and cognitive variables. In fact, an inclusion of latent variables can significantly reduce measurement errors and increase the reliability of the estimates (Kline, 2004). The small sample size might result in a lack of power to detect weak but significant relations. For instance, in this study, the insignificant relation between Grade 1 reading and Grade 3 reading (with a regression weight of 0.17) might be due to the lack of power. We surmise that if we have a larger sample, this relation may be significant although weak.

Thus, future replications with a larger sample size and latent variables for language and cognitive skills are needed.

## Data Availability Statement

The datasets presented in this study can be found in online repositories. The names of the repository/repositories and accession number(s) can be found at: “Data for: The Contributions of Language Skills and Comprehension Monitoring to Chinese Reading Comprehension: A Longitudinal Investigation,” Mendeley Data, V1, DOI: 10.17632/62mfct3gc8.1.

## Ethics Statement

Ethical review and approval was not required for the study on human participants in accordance with the local legislation and institutional requirements. Written informed consent to participate in this study was provided by the participants’ legal guardian/next of kin.

## Author Contributions

AZ: theory formulation, research hypothesis, data entry, analysis and interpretation of data, literature review, and Materials and Methods section. YG: theory formulation, research hypothesis, and Discussion section. SS and ML: analysis and interpretation of data and Results section. AB: research hypothesis and theory formulation. ML: Materials and Methods section. All authors contributed to the article and approved the submitted version.

### Conflict of Interest

The authors declare that the research was conducted in the absence of any commercial or financial relationships that could be construed as a potential conflict of interest.
